# Presence of skeletal banding in a reef-building tropical crustose coralline alga

**DOI:** 10.1371/journal.pone.0185124

**Published:** 2017-10-04

**Authors:** Bonnie Lewis, Janice M. Lough, Merinda C. Nash, Guillermo Diaz-Pulido

**Affiliations:** 1 Griffith School of Environment and Australian Rivers Institute–Coast & Estuaries, Griffith University, Nathan, Australia; 2 ARC Centre of Excellence for Coral Reef Studies, Queensland, Australia; 3 Australian Institute of Marine Science, Townsville, Australia; 4 Research School of Physics and Engineering, Australian National University, Canberra, ACT, Australia; National Taiwan Ocean University, TAIWAN

## Abstract

The presence of banding in the skeleton of coralline algae has been reported in many species, primarily from temperate and polar regions. Similar to tree rings, skeletal banding can provide information on growth rate, age, and longevity; as well as records of past environmental conditions and the coralline alga’s growth responses to such changes. The aim of this study was to explore the presence and characterise the nature of banding in the tropical coralline alga *Porolithon onkodes*, an abundant and key reef-building species on the Great Barrier Reef (GBR) Australia, and the Indo-Pacific in general. To achieve this we employed various methods including X-ray diffraction (XRD) to determine seasonal mol% magnesium (Mg), mineralogy mapping to investigate changes in dominant mineral phases, scanning electron microscopy–electron dispersive spectroscopy (SEM-EDS), and micro-computed tomography (micro-CT) scanning to examine changes in cell size and density banding, and UV light to examine reproductive (conceptacle) banding. Seasonal variation in the Mg content of the skeleton did occur and followed previously recorded variations with the highest mol% MgCO_3_ in summer and lowest in winter, confirming the positive relationship between seawater temperature and mol% MgCO_3_. Rows of conceptacles viewed under UV light provided easily distinguishable bands that could be used to measure vertical growth rate (1.4 mm year^-1^) and age of the organism. Micro-CT scanning showed obvious banding patterns in relation to skeletal density, and mineralogical mapping revealed patterns of banding created by changes in Mg content. Thus, we present new evidence for seasonal banding patterns in the tropical coralline alga *P*. *onkodes*. This banding in the *P*. *onkodes* skeleton can provide valuable information into the present and past life history of this important reef-building species, and is essential to assess and predict the response of these organisms to future climate and environmental changes.

## Introduction

Coralline algae are members of the phylum Rhodophyta (red algae), algae characterised by the presence of calcium carbonate (CaCO_3_) within their cell walls [1]. With a worldwide distribution and habitat ranging from the intertidal zone to over 250 m in depth [2], coralline algae are among the most abundant marine calcifying organisms to live in the euphotic zone [3]. The carbonate skeleton of coralline algae is primarily made up of high magnesium calcite (Mg-calcite), ranging from 10–20% mol MgCO_3_ calcite [4], meaning 10–20% of calcium in the calcite lattice has been substituted for Mg [5]. Other carbonate minerals such as aragonite [6, 7] and dolomite [8] are also present in the skeleton (via secondary precipitation).

Due to this precipitation of CaCO_3,_ coralline algae also play fundamental roles in temperate and tropical reef environments by cementing and stabilising the reef framework [9], providing habitat and food [9, 10], along with hard substrate and settlement cues for various invertebrate species such as corals [11, 12], and transferring carbon from the biological cycle to the geological cycle [3]. Although coralline algae have long been regarded as a vital component of healthy coral reef ecosystems [13], it is their high vulnerability to ocean acidification (OA) [14–16], and their function as palaeoenvironmental recorders [17–20] that has sparked renewed interest in coralline algae research. However, in order to properly understand the outcomes of OA research on coralline algae it is essential to first have knowledge of basic information, such as growth rates and mineralogy.

Growth rate is a vital attribute for any organism and is a metric used in population ecology that is fundamental to our understanding of population dynamics [21]. Longevity and age at reproduction are also key attributes (vital rates) for population dynamics. Currently there is little information available on growth rates of tropical coralline algae. Slow growth rates [22, 23] necessitating lengthy *in situ* studies in often difficult, and turbulent conditions such as found on reef crest environments (where highest reef accretion rates occur [24]) are partly to blame for this lack of basic information. With this in mind, our investigation explores the patterns of growth and banding in the skeleton of a dominant tropical reef-building coralline alga *Porolithon onkodes* and ascertains if these patterns can be used to determine past and present growth rates, without the need for lengthy *in situ* studies.

The distinct banding pattern observed in temperate and cold water coralline skeletons results from mineralogy changes in the mol% Mg of the precipitated CaCO_3_ [20], and/or structural changes in cell size and density [22, 25, 26]. These changes reflect seasonal variability in environmental conditions such as light and seawater temperature [10, 19, 26]. In regions of high seasonality, annual banding cycles are characterised by long, less densely calcified cells and high mol% Mg in summer, and by shorter more densely calcified cells and lower mol% Mg in winter [17, 20, 25], with the seasonal range in mol% Mg dependent on the species and location. For example, Halfar *et al*., [20] using electron microprobe analysis, found mol% MgCO_3_ ranged from 7.7 to 18.5 in *Lithothamnion glaciale* from Newfoundland, while *L*. *crassiusculum* from the Gulf of California ranged from 13.2 to 22.5. The varying composition of the carbonate skeleton is thought to be driven mainly by changes in temperature [27] with higher temperatures favouring Mg substitution in calcite. In recent years, interest in skeletal banding of coralline algae as a method for determining growth rates and as archives of paleoclimatic information has produced many studies, the majority of which have been conducted in the colder climates of northern Europe and Canada [28] (Table 1). Skeletal banding studies in tropical waters are few, however, Agegian, [29], Darrenougue *et al*., [26] and Sletten *et al*., [30] all observed density banding in branching or rhodolith species from the Hawaiian Islands, New Caledonia and the Gulf of Panama respectively. Darrenougue *et al*., [26] also observed seasonal changes in the Mg/Ca ratio of the rhodolith species *Sporolithon durum*. The presence of banding in these tropical studies suggests that the method of using skeletal banding to determine growth rates may also be successfully applied to coralline algae of the Great Barrier Reef (GBR), where average temperatures remain high (average winter and summer temperatures in the southern GBR are 21.8°C and 26.7°C, respectively [31]. Importantly, examining skeletal banding will help us obtain baseline demographic information, without the need for long-term *in situ* growth experiments, for the most important and abundant reef-building crustose coralline alga (CCA) in the GBR, *P*. *onkodes* [32, 33]. Once established, this method can potentially be applied to determine growth rates of *P*. *onkodes* across the entire region of the GBR simply by examining these banding patterns, making this growth information more accessible and attainable.

**Table 1:** Coralline algae skeletal banding studies. Coralline algae reported to have skeletal banding patterns that have been used to measure growth or as a palaeoenvironmental record.

Species	Growth Form	Region	Banding Type	Reference
*Clathromorphum compactum*	Encrusting	Subarctic–Labrador, Canada	Mg content	Adey *et al*., [34]
		Subarctic–Newfoundland, Canada	Mg content	Gamboa *et al*., [35]
		Subarctic /Temperate–Gulf of Maine, US	Stable oxygen isotope	Halfar *et al*., [36]
		Subarctic–Northwestern Atlantic	Mg content	Halfar *et al*., [37]
*C*. *nereostratum*	Encrusting	Subarctic–Bering Sea	Oxygen isotope	Halfar *et al*., [38]
		Subarctic–Aleutian Islands, Bering Sea	Mg content and oxygen isotopes	Hetzinger *et al*., [39]
*Lithothamnion crassiusculum*	Rhodolith	Temperate/Sub tropical—Gulf of California, US	Carbon isotope	Frantz *et al*., [40]
		Temperate/Subtropical: Gulf of California	Mg content	Halfar *et al*., [20]
*L*. *glaciale*	Rhodolith	Temperate–West coast of Scotland	Density	Burdett *et al*., [18]
		Arctic–Northern Norway	Density	Freiwald and Henrich, [25]
		Subarctic–Newfoundland, Canada	Oxygen isotope and Mg content	Halfar *et al*., [20]
		Temperate—West coast of Scotland	Mg content	Kamenos *et al*., [5, 17]
		Temperate—West coast of Scotland	Density	Kamenos and Law, [19]
		Temperate—Kattegat, Norway	Mg content, cell size	Ragazzola *et al*., [41]
*L*. *muelleri*	Rhodolith	Temperate/Subtropical–Gulf of California, US	Density	Rivera *et al*., [23]
*3 species–Lithothamnion sp*, *Lithophyllum sp*, *Lithoporella sp*	Encrusting, Branching & Rhodolith	Tropical–Gulf of Chiriqui, Gulf of Panama, Pacific Ocean	Mg content and density	Schafer *et al*., [42]
*Phymatolithon calcareum*	Rhodolith	Temperate–Northern Ireland	Density and cell size	Blake and Maggs, [22]
		Temperate–West coast of Scotland	Mg content	Kamenos *et al*., [5, 17]
*Porolithon gardineri*	Branching	Tropical–Hawaii	Density and cell size	Agegian, [29]
*Porolithon onkodes*	Encrusting	Tropical–Great Barrier Reef, Australia	Mg content and density	This paper
*Sporolithon durum*	Rhodolith	Tropical–New Caledonia	Density, cell size and Mg content	Darrenougue *et al*., [26]

**Fig 1:**
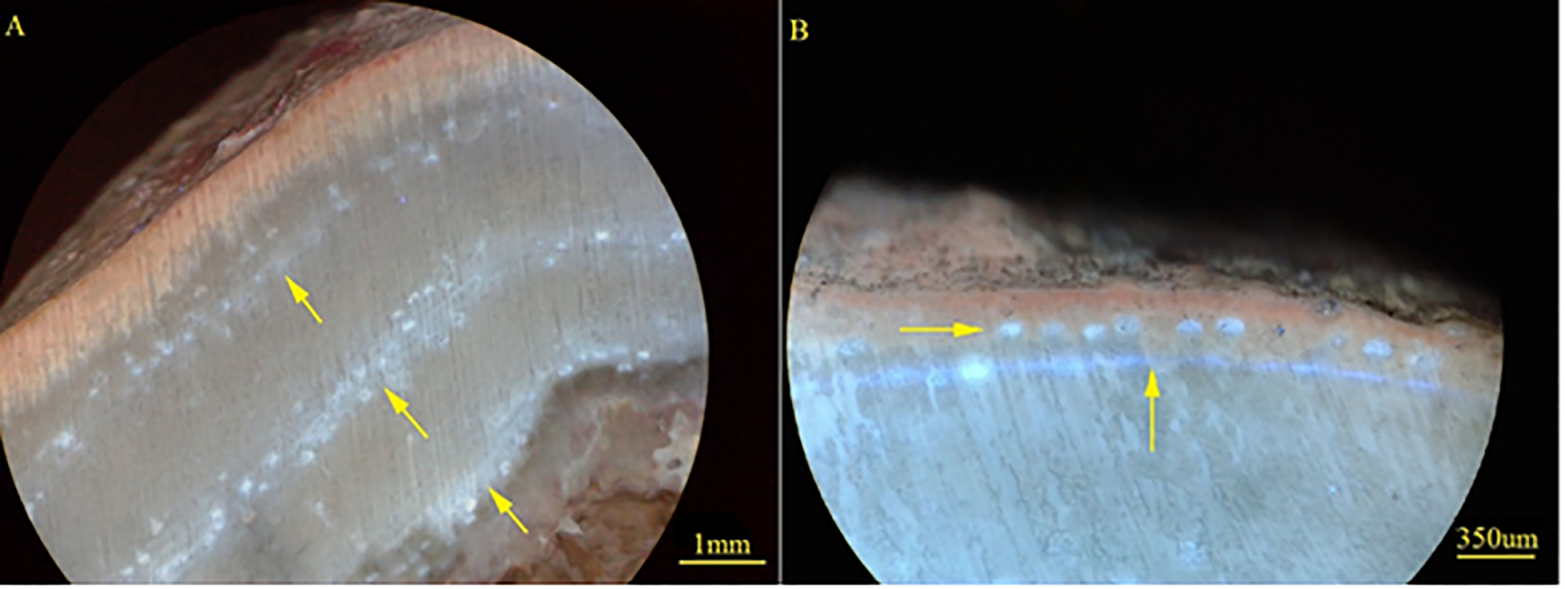
Conceptacle banding of *Porolithon onkodes* under UV light; **a.** Cross-section of *P*. *onkodes* under UV light (BX50 Olympus microscope), arrows point to bands of conceptacles in skeleton made up of two or more conceptacles rows on top of each other; **b.** Cross-section of *P*. *onkodes* under UV light, horizontal arrow point to conceptacles, vertical arrow points to stain mark indicating the start of the summer season.

Conceptacle banding is another feature from which growth rates can be determined for coralline algae [43]. All species of coralline algae form reproductive chambers, known as conceptacles, that are open to the surface and contain reproductive structures [44, 45]. While information on coralline reproduction cycles are largely unknown on the GBR, in temperate regions coralline algae have distinct reproductive cycles, and the time of year these reproductive structures are formed is largely species specific [46, 47]. With continued growth these rows of buried conceptacle chambers form bands of newly regenerated or infilled cells, often with a different mineralogical composition from the surrounding skeleton [7, 8]. In our study, these changes in mineralogy or organic content in the chambers fluoresce under UV light, producing discernible horizontal banding in the coralline crust, as shown in Fig 1A. Once calibrated to seasonal or annual timeframes, conceptacle banding could also be used as a method for obtaining demographic information such as growth rate, age and longevity of tropical coralline algae.

The aim of this study was to determine the presence of skeletal banding in the abundant and tropical reef-building species *P*. *onkodes* on the GBR, and to establish whether this skeletal banding can be used to determine current or past growth rates. To do this we used a range of well-established as well as novel mineralogical techniques. Specific questions addressed in our study include:

■ Do seasonal changes in mol% MgCO_3_ exist in *P*. *onkodes*? This was determined using the standard XRD method [48].
■ Does the reproductive cycle of *P*. *onkodes* form annual conceptacle banding? This was determined using the novel method of observing this banding under UV light.
■ Are bands of short, heavily calcified cells with low mol% MgCO_3_, and long, less calcified cells with a high mol% MgCO_3_ present in the skeleton? This was analysed using X-radiography and the recently utilised (in coralline algae research) micro-CT scanning [49, 50].
■ Do changes in mol% Mg form banding patterns throughout the coralline algae skeleton when traversing from the pigmented (photosynthetic) surface to the unpigmented base (hypothallus) on the algae? This was analysed using a new method, Quantitative Evaluation of Minerals by Scanning electron microscopy (QEMSCAN), which can determine changes in mineral composition of the skeleton.

## Materials and methods

### Sample collection and staining

**Fig 2:**
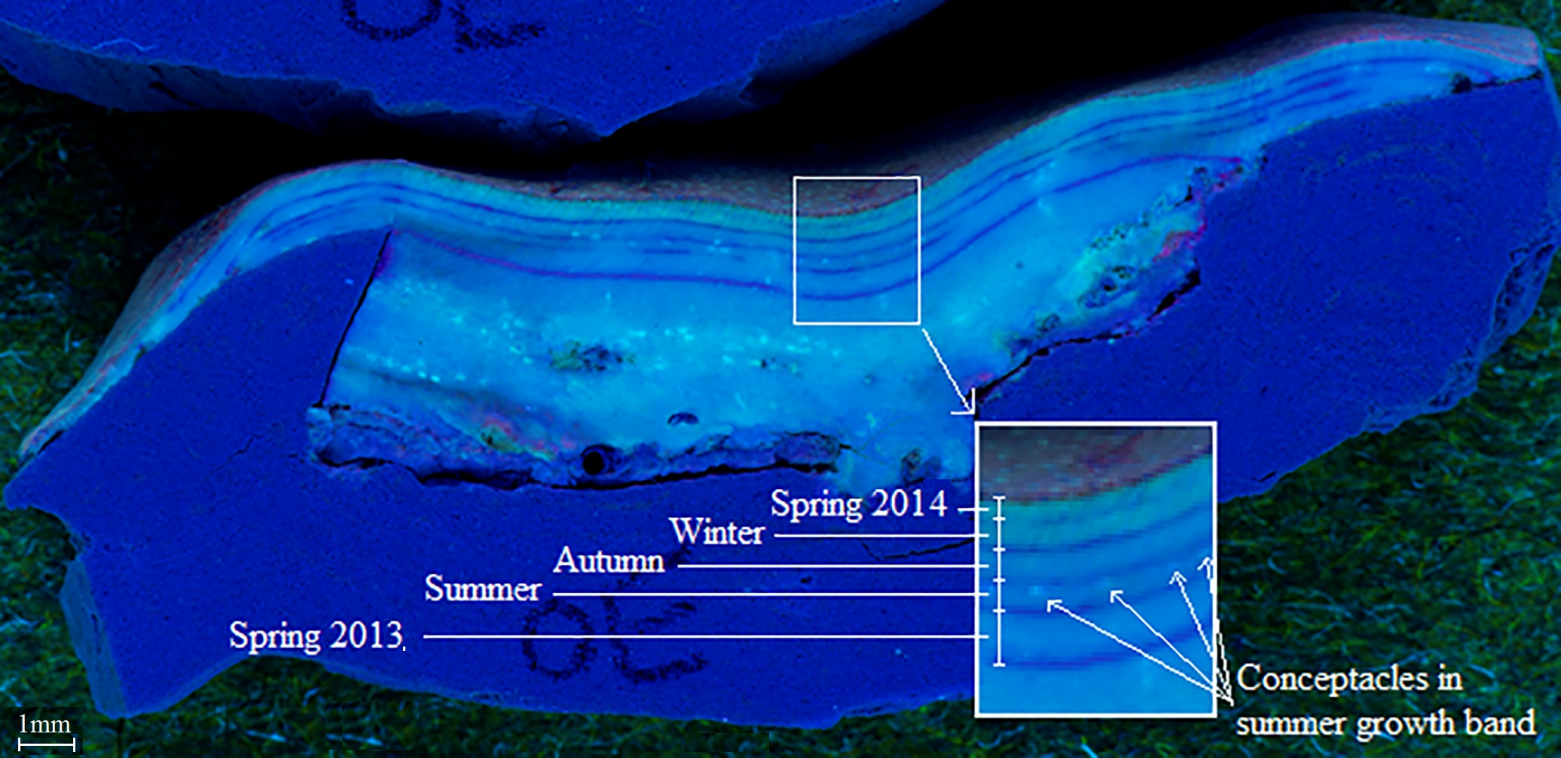
*Porolithon onkodes* long-term sample. Long-term sample (OE) viewed under UV light showing five alizarin stains, each stain line represents the start of a season, beginning with Spring 2013 the lowest stain mark on the skeleton; conceptacles are also present within the summer growth band.

Fieldwork for this study was conducted at the Tenements 1 site (23°26’00.4 S, 151°55’41.3 E) of Heron Island in the southern GBR, Australia under the permission of the Great Barrier Reef Marine Park Authority (permit number G13/36022.1). Samples (S1 Table) were collected from the reef, stained in the laboratory (see below, [51]), deployed back on the reef and finally collected during five separate three-month sampling periods, representing the different climatic seasons from austral spring 2013 to summer 2014. This seasonal sampling allowed for the determination of exact time periods for which reproductive conceptacles appeared, and the calculation of seasonal growth and calcification rates [51]. At the beginning of each season 20 specimens of *P*. *onkodes* were carefully collected using hammer and chisel from the nearby reef crest/upper reef slope (< 6 m) of Tenements 1, and transported back to the outdoor flow-through tank facilities on Heron Island Research Station (HIRS), where seawater was supplied directly from the reef lagoon. Samples were cleaned by hand to remove epiphytes, invertebrates and loose material and cut to size (3 x 3 cm chips). After cutting, samples were stained using the Alizarin Red stain at a concentration of 0.25 g L^-1^ for 24 hrs [22, 52, 53] and set into epoxy rings to secure the sample and prevent potential dissolution of exposed skeleton (details of the methods are found in [51]). The epoxy rings were made of 40 mm PVC pipe filled with Selleys Aqua Knead It ™ epoxy, set inside a previously prepared 90 mm PVC ring filled with cement and covered with a thin layer of the epoxy to keep a uniform surface of the same substrate. Once prepared, samples were attached to galvanised racks (2 per rack) and secured to the reef slope (S1 Image) at approximately 5–6 m depth (high tide). Following the seasonal three month period all 20 samples were retrieved, cleaned of fouling organisms and oven dried at 60°C for 24 hrs. These samples were replaced on the reef by 20 newly collected and stained samples for the following season. Five long-term samples were deployed for the entire 15 month experiment. These samples were retrieved from the reef briefly for staining at the beginning of each season then immediately redeployed providing a continuous record over the 15 month experiment. Each of these samples produced five alizarin marks defining the growth area of the five seasons (Fig 2). For subsequent analyses all samples were cross-sectioned (approx. 3 mm thick) using a dremel diamond cut wheel. In total 100 seasonal samples (5 sampling periods, 20 CCA fragments/period), and five long-term samples were deployed (see S1 Table).

### UV imaging to examine conceptacle banding

Conceptacle banding was examined in both seasonal and long-term samples (S1 Table) using fluoroscopy microscopy and camera images. This technique allows visualisation of distinct conceptacle banding patterns in the skeleton when illuminated under UV light, as displayed in Fig 1A and 1B, which then may be calibrated and used to measure growth rates. The presence of this fluorescent banding was revealed when analysing alizarin stained samples under UV light. The alizarin staining allowed for the frequency of conceptacle banding formation in the *P*. *onkodes* skeleton to be determined without affecting the UV conceptacle banding observations. Although not previously applied to coralline algae, UV banding has been used in massive corals to reconstruct past river flow and rainfall [54–56]. These UV bands are formed by the inclusion of terrestrial humic substances into inshore corals exposed to river flood plumes [55].

**Fig 3:**
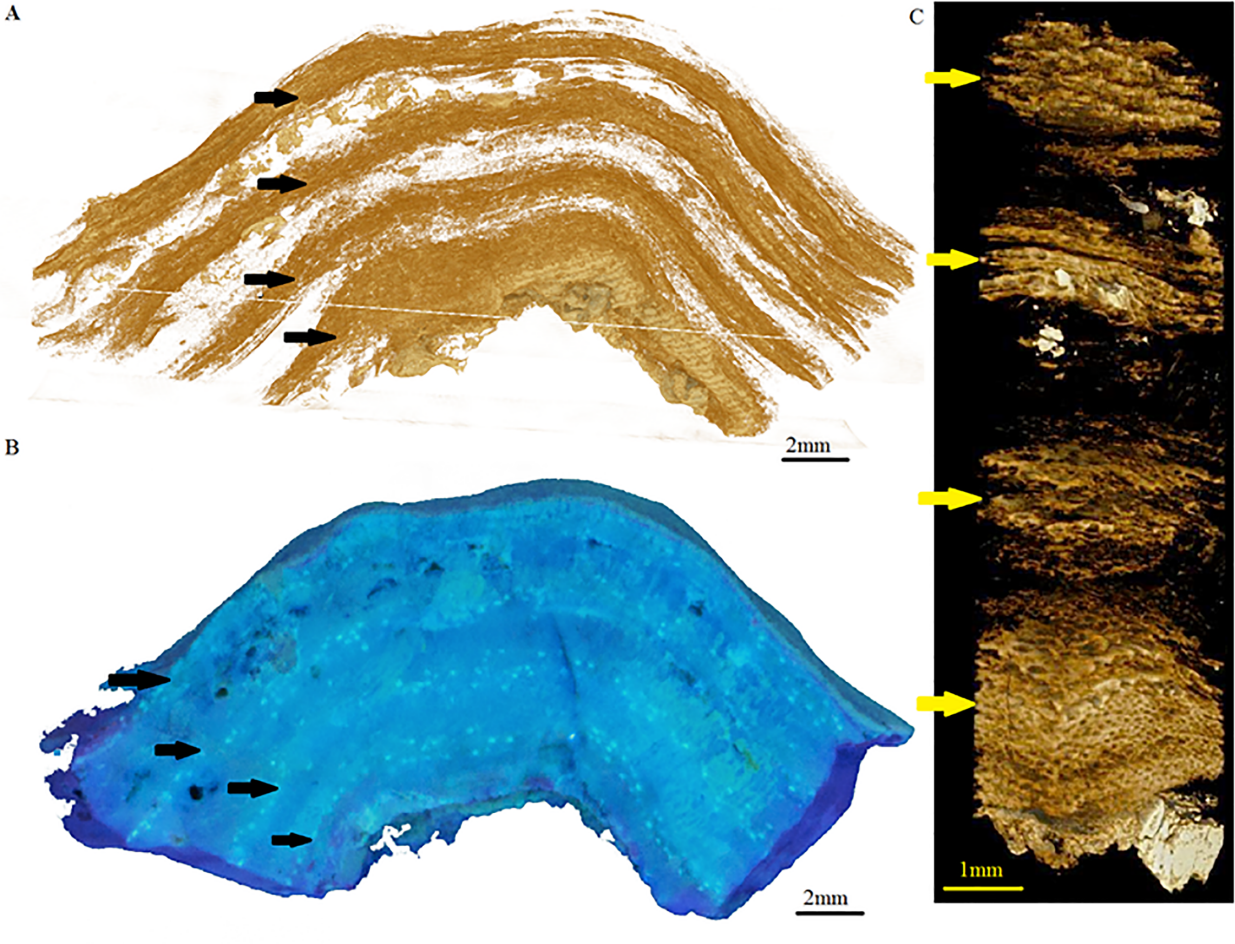
*Porolithon onkodes* density and conceptacle banding. Three images of the same *P*. *onkodes* sample (PK) showing relationship between conceptacle and density banding, arrows indicate areas of a) high density banding (dark areas) in 2D micro-CT image; b) conceptacle banding in UV image; c) high density banding (in light areas) in 3D micro-CT image.

Initial images were taken using a BX50 Olympus fluorescent compound microscope under a UV filter (Fig 1A and 1B). To capture the entire cross-section a Nikon D800e DSLR camera with a Nikon 105 mm micro lens was used (Fig 3B). Exposure was set to 200ISO, F22, 5 secs and the light source was from 6 x 40W BLB UV Fluorescent tubes in a purpose-built box. Images were post processed using Adobe Photoshop to control colour balance and contrast. Growth measurements were not taken where signs of damage/alteration (cavities, endolithic algae, etc.) were evident.

### X-ray imaging and Micro-CT scans to examine density banding

X-radiograph (X-ray) imaging analysis was conducted at a resolution of 79 pixels/cm using twelve seasonal (3 samples per season), and one long-term sample at the Australian Institute of Marine Science (AIMS, Townsville, Eric Matson). While not yet widely applied to coralline algae, X-ray imaging is a commonly used technique for determining the presence of annual banding in corals (as reviewed by [57]). These bands are derived from seasonal changes in skeletal density, which can then be used to determine linear extension rates [57]. Micro-CT scanning was conducted after banding was observed from the X-ray analysis. Samples were sent to the Australian National University (ANU, Department of Applied Mathematics, Dr Michael Turner and Dr Levi Beeching) for higher resolution scanning under the micro-CT, allowing for more precise measurements of banding width and extension rates.

Five seasonal and one long-term sample of *P*. *onkodes* were sent to the ANU (Centre for Advanced Microscopy) for 3D imaging. Scans were taken using a high-cone-angle helical micro-CT system at a resolution of 8.1 microns, with a 200 kV reflection-style X-ray source and large area amorphous-silicon (a-Si) flat panel detector. The open source computer software program Drishti (developed at ANU) was employed to render and analyse the image stacks. This method of micro-CT scanning to analyse density in the coralline skeleton was first used by Torrano-Silva *et al*., [50], followed more recently by Krayesky-Self *et al*., [49].

### Mineralogy mapping to determine Mg-calcite banding

Mineralogical composition mapping of a single long-term *P*. *onkodes* skeleton was also carried out at ANU (Centre for Advanced Microscopy, Dr Frank Brink) using QEMSCAN. This mapping integrates scanning electron microscopy and energy-dispersive X-ray spectroscopy (SEM-EDS) hardware with software to generate micron-scale compositional maps of rocks and sediments and is widely used in mining and petroleum industries [58]. To apply this method of mineral analyses to the MgCO_3_ coralline algae skeleton, the technique was modified to determine changes in the Mg intensity (i.e., high Mg-calcite, dolomite and magnesite), rather than changes between minerals. Images of these changes in Mg intensity were taken at a resolution of 5 μm. SEM-EDS, using a Zeiss Ultraplus field emission scanning electron microscope, operated at 15.0 kV, 10.9 mm working distance was then used to provide elemental composition using spot analysis within each of these bands. Samples were carbon coated and mounted using carbon tape. As outlined in Nash *et al*., [8, 48], Mg-calcite was identified ranging from 8–25 mol% MgCO_3,_ dolomite as 38–62%, and magnesite as >80% (however measurements between 62–99% are thought to be magnesite with small amounts of the neighbouring Mg-calcite or dolomite).

### SEM to examine cell size and density banding

To determine the presence of banding in the *P*. *onkodes* skeleton, due to changes in cell size and skeletal density, SEM was employed. Cross-sectioned samples were prepared for SEM by lightly polishing the sample by hand using grit sandpaper. SEM was carried out at ANU (Centre for Advanced Microscopy) using a Hitachi 4300 SE to produce backscatter electron images at a 27.1 mm working distance operated at 15.0 kV. SEM-EDS was also carried out (as outlined above) to determine elemental composition in these bands.

### X-ray Diffraction (XRD) to determine seasonal mineralogy

Powdered XRD analysis was used to determine seasonal changes in mol% MgCO_3_ of the *P*. *onkodes* skeleton from four of the five sample seasons (S1 Table). Average seasonal seawater temperatures were taken from Lewis *et al*., [51]. Pigmented tissue was scraped from the surface of the sample (no white crust was included), and ground into a powder by hand using mortar and pestle. The MgCO_3_ was determined following methods in Nash *et al*., [48]. The presence of dolomite was checked for using the peak asymmetry method. This method uses the asymmetry off the right side (higher 2-theta) of the Mg-calcite XRD peak to detect dolomite. A shoulder off the higher 2-theta side of the peak indicates that magnesite (MgCO_3_) is also present. This asymmetry and shoulder is captured with the asymmetry mol% measurement. The asymmetry mol% is used to compare differences in relative dolomite and magnesite quantities. A one-way ANOVA with Welch test was used to determine any significant differences between season and mol% MgCO_3._ Data normality and homogeneity of variance were tested using Shapiro-Wilk and Levene’s test, respectively. Games-Howell test was used for post-hoc comparisons. Linear regression analysis was used to further analyse the relationship between mol% Mg and seawater temperature.

## Results

### UV imaging to examine conceptacle banding

The alizarin mark pinpointed the area of growth in the skeleton that formed during a particular season, allowing for the determination of exact time periods during which the formation of conceptacles occurred (Fig 2). For *P*. *onkodes*, conceptacles were predominately observed over the summer season (Figs 1B and 2), occurring once a year. We occasionally observed sporadic (1 or 2) individual conceptacles appearing immediately before or after the summer season. The bands of annual conceptacles were the dominant feature of the *P*. *onkodes* skeleton when examined under UV light (Fig 1A). Annual growth rates of 1.38 (±0.03) mm year^-1^, determined by the alizarin stain and calculated in Lewis *et al*., [51] using seasonal means (*n* = 20), coincided with this conceptacle banding of 1.48 (±0.1) mm year^-1^ (*n* = 10), with the distance measured from the middle of one conceptacle band to the middle of the next conceptacle band.

### Micro-CT scans to examine density banding

Evidence of density banding was clearly seen in the X-ray images (S2 Image) however the resolution (79 pixels/cm) was too coarse to clearly define and measure this banding. Under micro-CT scanning, with a resolution of 8 μm (as presented in Fig 3A and 3C), density banding was clearly evident and well defined in the *P*. *onkodes* skeleton. Under this high-resolution scanning, conceptacles could also be observed in the skeleton, and the thickness of the bands and distance between banding could be measured. From these images and measurements it was also observed that changes in skeletal density appeared to coincide with conceptacle bands in the skeleton (Fig 3B, S3 Image), where conceptacles are usually present in the high density bands.

### Mineralogy mapping to determine Mg-calcite banding

**Fig 4:**
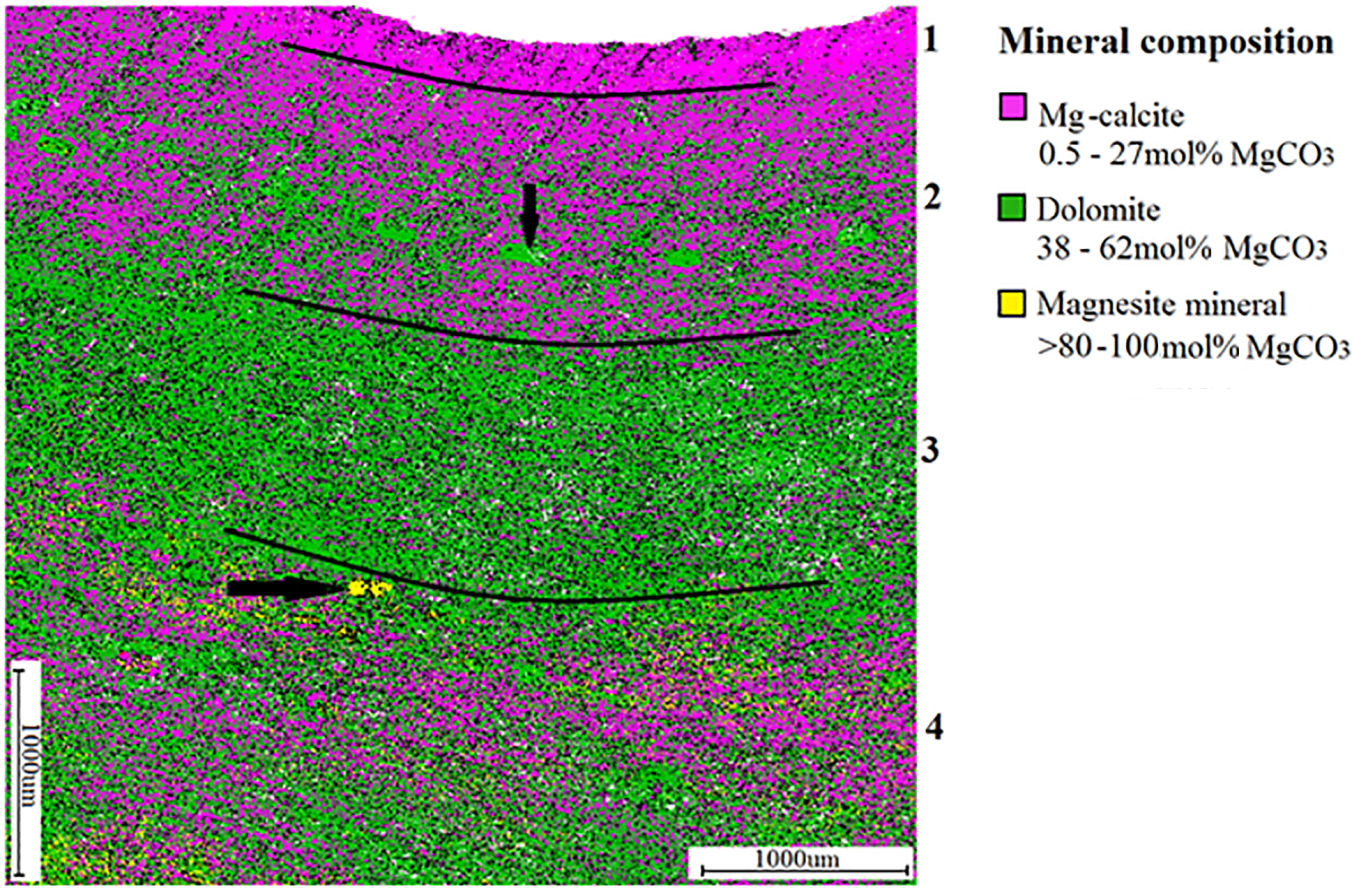
QEMSCAN of *Porolithon onkodes*. Mineralogical mapping using Quantitative Evaluation of Minerals by Scanning electron microscopy (QEMSCAN) showing four bands (labelled on right side of image) of dominant mineral changes in *P*. *onkodes* from Heron Island, Great Barrier Reef. Conceptacles are indicated by arrows. Black lines indicate changes in dominant mineral.

**Fig 5:**
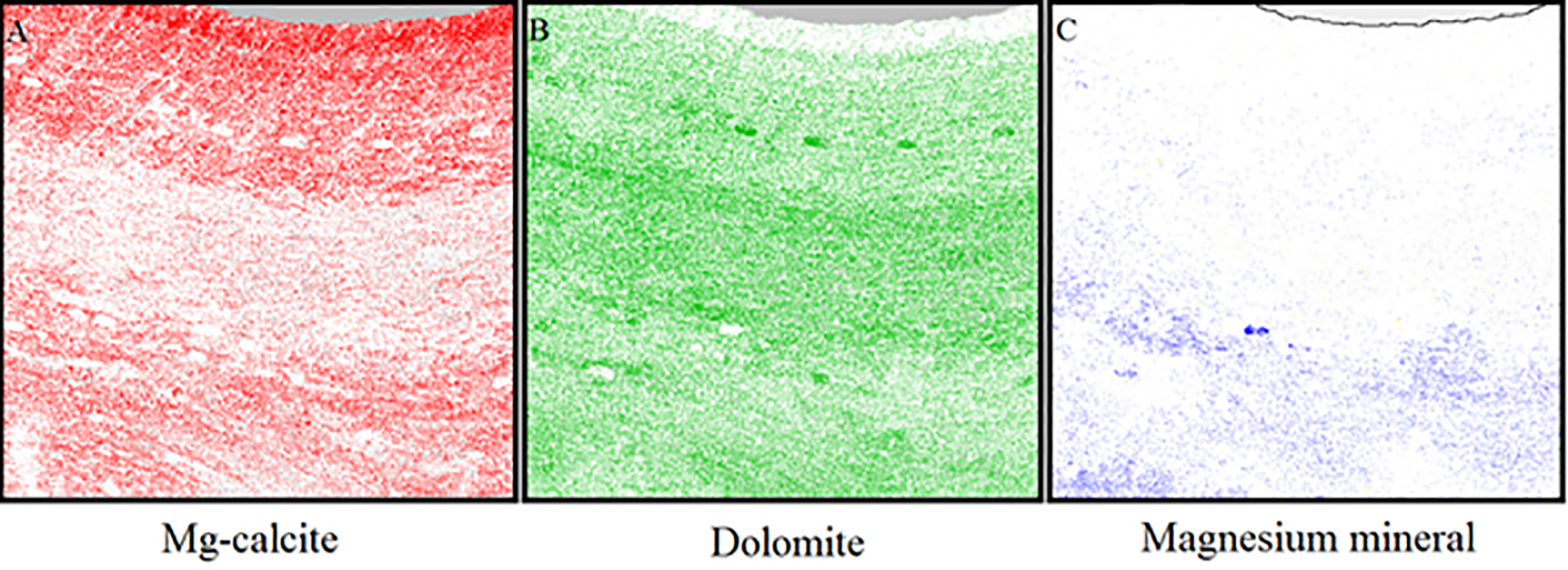
Mineralogy mapping of *Porolithon onkodes* skeleton. Individual QEMSCAN images of dominant mineral compositions, the intensity of the colour reflects the intensity of the mineral a) Mg-calcite; b) Dolomite; c) Magnesite mineral.

Images of the long-term sample (S1 Table) taken with the QEMSCAN revealed bands of change in the abundance of each mineral moving from the epithallus down to the base of the skeleton, i.e., the hypothallus (Figs 4 and 5). The first band, starting at the epithallus to ~ 300 μm in depth (shown as Band 1 in Fig 4), was composed of densely calcified high Mg-calcite, with an average of 16 mol% MgCO_3_. Below this, the second band (starting at ~ 300 μm and running down to ~1430 μm depth) was also dominated by high Mg-calcite, however the mineral dolomite was also present in this band. Using SEM, this dolomite (~ 60 mol% MgCO_3_) was found to occur in the cell lining and around the edges of infilled conceptacles of this high-Mg band. In the third band (starting at ~ 1430 μm and ending at ~ 2860 μm depth) the dominant mineral changed from high Mg-calcite to dolomite with ~ 60 mol% MgCO_3._ Although difficult to image with SEM (due to damage caused by QEMSCAN) this increase in dolomite may be due to thicker cell linings containing dolomite within this band. The fourth and final band of mineralogy (starting at ~ 2860 μm and running down to the bottom of the skeleton) contained high Mg-calcite, dolomite and small quantities of the magnesite mineral, identified as > 80 mol% MgCO_3_ using SEM spot analyses.

### SEM to examine CaCO_3_ carbonate and cell size banding

**Fig 6:**
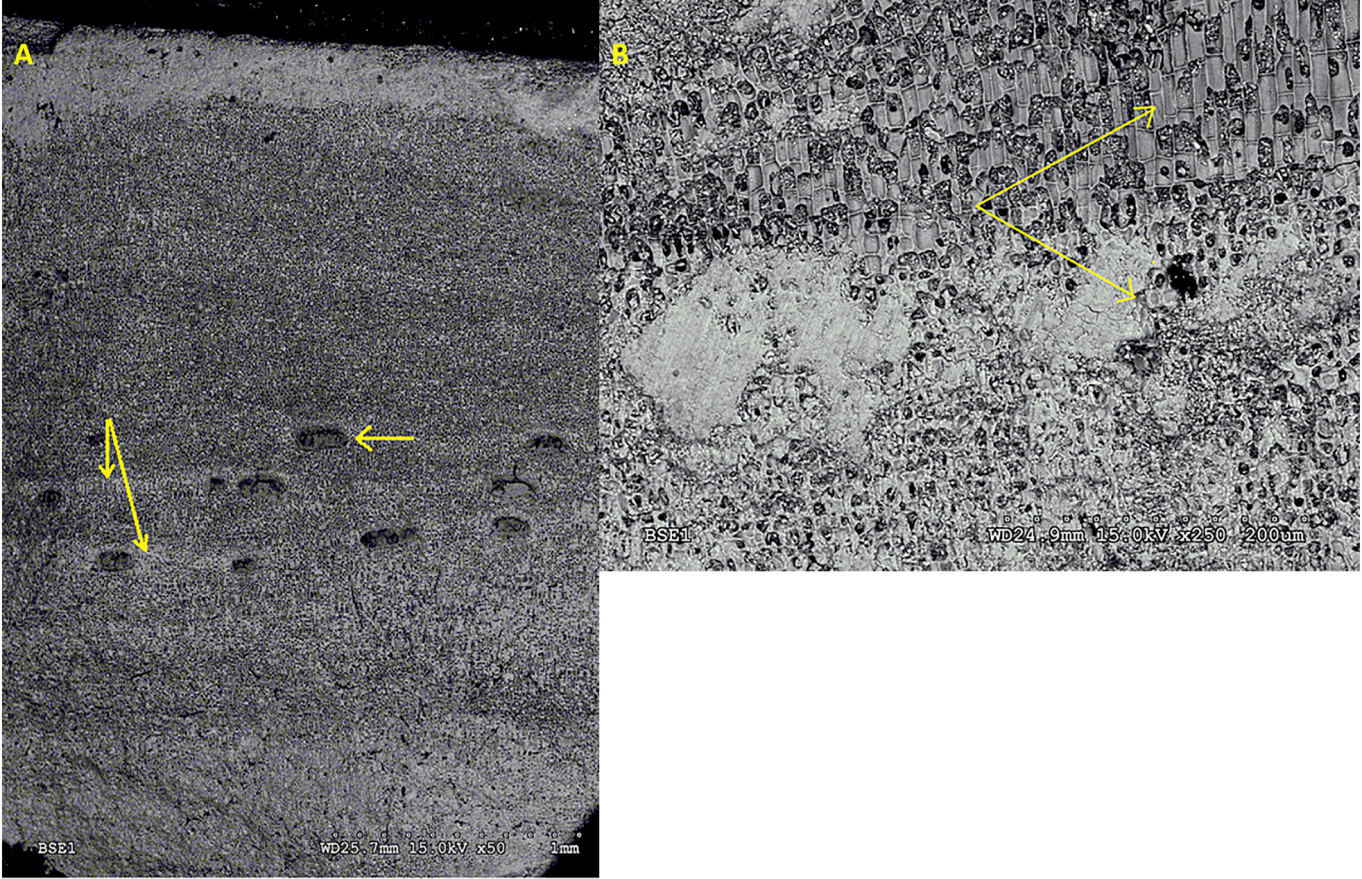
**a**. **SEM of *Porolithon onkodes*** cross-section showing no regular banding patterns (from changes in cell size) as seen in other coralline algae samples (e.g., as shown in [17]). Vertical arrows indicate areas of higher density within the conceptacle band, horizontal arrow indicates conceptacle, **b**. **Rapid growth cells in *Porolithon onkodes***. Long, less calcified cells in *P*. *onkodes* skeleton on the top right hand corner of image (upper arrow) indicate an area of rapid growth compared to cells beneath this (lower arrow); in this sample the rapid growth overgrew the epoxy that had covered that part of the epithallus.

SEM analysis of seasonal *P*. *onkodes* samples did not reveal the obvious banding pattern in the skeleton (Fig 6A) seen in many other species that result from changes in cell size between summer and winter seasons or from seasonal variations in Mg-calcite composition. Changes in cell size and density were observed in the skeleton, however these were small areas of long, less densely calcified cells that were probably rapidly growing [19, 25], as shown in Fig 6B.

### X-ray Diffraction (XRD) to determine seasonal mineralogy

**Fig 7:**
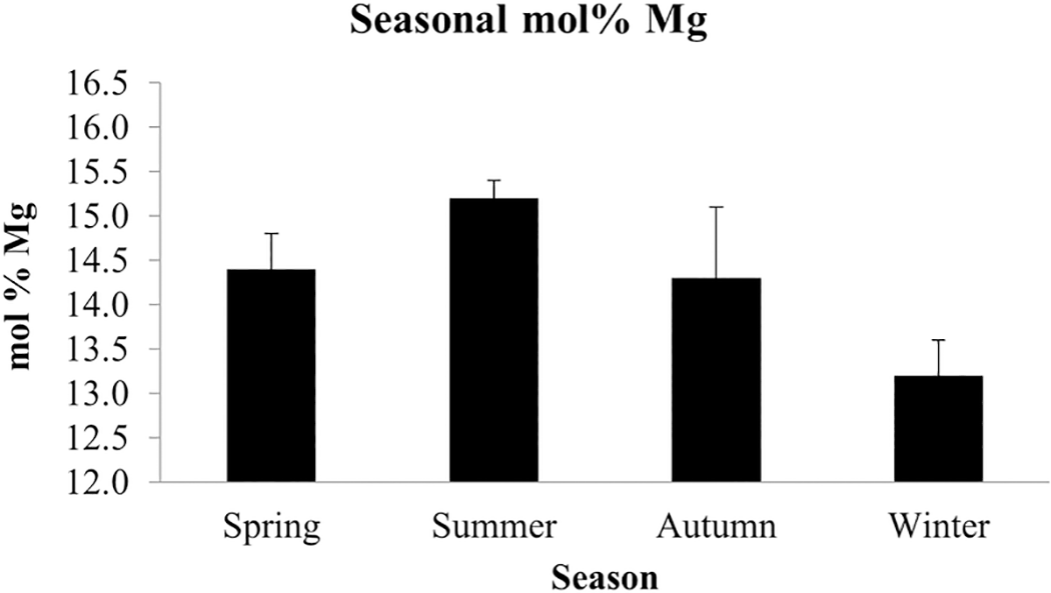
*Porolithon onkodes* seasonal mol% Mg. XRD analysis showing the seasonal variation in the mol% Mg of *P*. *onkodes* from the Great Barrier Reef. Values are means ± SD, *n* = 5

XRD analysis carried out on the pigmented surface tissue of five *P*. *onkodes* samples from each of the four (of the five) seasons (spring 2013 –summer 2014) revealed seasonal changes in the mol% MgCO_3_ (Fig 7). Average mol% MgCO_3_ was highest in summer (15.2 ± 0.2) with declining values in spring (14.4 ± 0.4) and autumn (14.3 ± 0.8) and lowest values in winter (13.2 ± 0.4). A significant difference was found in the mean of mol% MgCO_3_ between seasons (ANOVA, *f*_3, 8.4_ = 30.96, *p* < 0.001). No dolomite was detected in these surface tissue samples. Post hoc comparisons (Games-Howell) showed that the seasonal mineralogy differences were between summer and winter (*p* < 0.001), summer and spring (*p* = 0.016), and spring and winter (*p* = 0.007). To explore the relationship between the average temperature recorded in each climatic season and the average mol% Mg quantified in our samples, we conducted linear regression analysis. The analysis showed a positive relationship between these two variables (linear regression analysis (last squares), R^2^ = 0.957, *n* = 4, *p* = 0.022).

## Discussion

This is the first study documenting the presence of growth and conceptacle bands in an important reef-building crustose coralline algae of the GBR using a variety of skeletal and mineralogical techniques. UV imaging, X-rays, micro-CT scans, and mineralogy profiles provided strong evidence of the presence of skeletal bands that are related with the environmental seasonality to which the CCAs are exposed. Skeletal bands can be used to determine rates of growth, reproductive periodicity and mineralogical variation. Importantly, calibration of these bands with known seasonal variability will allow a better understanding of the potential effects of the environmental changes projected for the future on the growth and calcification of important reef building coralline algae.

### UV images to examine conceptacle banding

**Fig 8:**
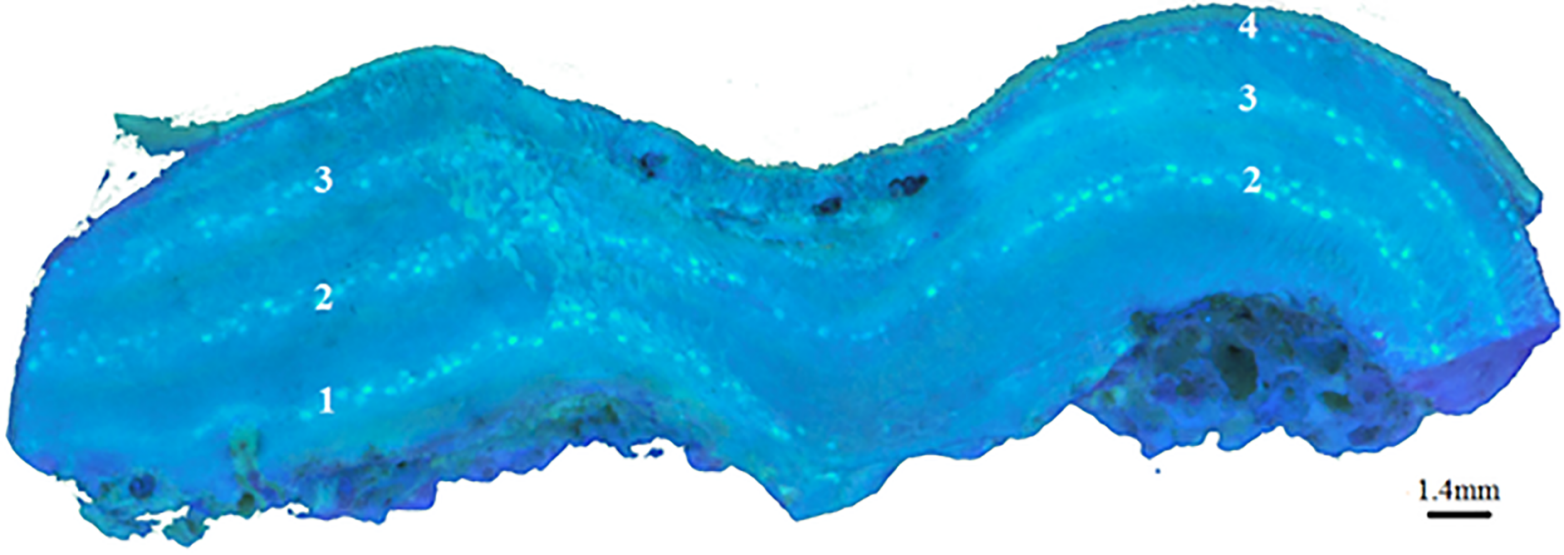
*Porolithon onkodes* conceptacle banding. UV image of *Porolithon onkodes* (NV) showing four bands of conceptacles (represented by the four horizontal bands of white dots) indicating age and growth rate of the sample. The growing edge is located at the top of the sample.

Conceptacle banding was clearly identifiable and uniform in the *P*. *onkodes* skeleton, displaying great potential for use as a proxy in the determination of past and present growth rates in tropical coralline algae. As little information is available regarding reproductive cycles of coralline algae on the GBR (e.g., [45]), before growth rate could be determined using this conceptacle banding, the frequency at which the banding occurs first needed to be established. By marking each season with alizarin it was observed that *P*. *onkodes* produce conceptacles once a year, predominantly during the summer season. The measured distance between this banding gave an annual vertical growth rate of 1.48 (±0.1) mm year^-1^, and the number of bands in the skeleton indicated the age of the organism. The period of time and growth that occurred before the earliest set of conceptacles formed is unknown. However, *P*. *onkodes* have been observed to start producing conceptacles within 3 months of settlement as observed and illustrated in Ordonez *et al*., [59], (Image S.2.B), therefore it is likely reproduction starts during the first summer after settlement. With the periodicity of conceptacle banding in the *P*. *onkodes* skeleton established, this inexpensive and efficient method for determining growth rate can be applied to determine growth rate of *P*. *onkodes* at different spatial scales across the GBR. For example, Fig 8 presents a *P*. *onkodes* sample collected from the southern GBR; this image shows four clearly distinguishable conceptacle bands in the crust representing four years growth. The distance between the conceptacle banding gives a growth rate of 1.4 mm year^-1^ and the relatively uniform banding indicated this growth rate was consistent over the four years and that reproduction occurred annually without disruption.

### Density and cell size banding

Seasonal banding, often used for calibrating growth rate and caused by changes in cell length and density [22] was not evident in the *P*. *onkodes* skeleton under SEM analysis (Fig 6A). Areas of rapid growth, represented by longer, less calcified cells were present in the *P*. *onkodes* crust; however these occurred in small areas such as in the cell regrowth of damaged tissue or (as in the case of Fig 6B) to grow over the epoxy shading the epithallus, and were not consistent across the width of the sample suggesting they are not a response to seasonal environmental changes. Previous studies on *P*. *onkodes* using SEM imaging have also not noted any cell length banding [60, 61]. This lack of banding due to changes in cell length may be due to the relatively small seasonal temperature range in tropical environments.

Although density banding was not clear in the SEM, it was evident using micro-CT imaging. Density banding in the tropical *P*. *onkodes* appears to be strongly related to the conceptacle bands (Fig 3). In this figure, the three images of the same sample show areas of high density banding associated with the presence of conceptacles (in summer) and areas of low density banding in areas where conceptacles were absent. This pattern of higher density bands in summer is in direct contrast to banding reported in areas of high seasonality, where highest density was observed over winter [19, 25]. While further analysis is needed to determine the cause of these areas of higher density within the conceptacle bands, it may be due to the infilling of aragonite and dolomite into the conceptacle cavities [7, 8] as shown in Fig 6A. Additionally, the density may be influenced by the cells surrounding the conceptacle cavity that have become compressed during conceptacle formation or, as shown in Fig 6A, from cells with more dolomite or magnesite infill that appear within the conceptacle banding area.

The method for measuring growth using density or conceptacle banding is very similar, based on measuring the distance between the density or conceptacle bands. Therefore the precision of these two methods is also similar and the results we obtained from these two methods were the same. In species where traditional methods such as Mg/Ca ratios or changes in cell size and density are not well defined, conceptacle banding under UV light may prove to be an efficient and cost-effective method of establishing coralline growth information.

### Mineralogy mapping to determine Mg-calcite banding

Maps of the mineral make-up of *P*. *onkodes* using the QEMSCAN revealed bands in the skeleton resulting from changes in Mg abundance (Figs 4 and 5). To confirm changes in the elemental composition within each of these identified bands, SEM-EDS analysis was applied. The top band (Fig 4) of pigmented tissue at the surface (epithallus) of the skeleton revealed a mol% MgCO_3_ consistent with that found in the seasonal XRD analysis of 13–15%. This epithallus band of new growth contained very little or no dolomite (Fig 5), a finding consistent with Diaz-Pulido *et al*., [7] and Nash *et al*., [61], who both reported dolomite mainly in the perithallus part of the skeleton (i.e. hypothallus and perithallus that lack pigmented algal tissue) of *P*. *onkodes*. Below this, a band of purely high Mg-calcite (Band 2) begins, distinguished from ‘Band 1’ by the presence of dolomite. ‘Band 3’ was an area dominated by the dolomite mineral, and ‘Band 4’ was distinguished by the appearance of the mineral magnesite. The increasing presence of the minerals dolomite and magnesite from the surface down to the base of the skeleton has not been previously reported. A study by Nash *et al*., [8] noted a ‘patchy trend for increasing cell in-fill towards the base’ however only the top part of the crust was observed in that study and the process driving the formation of these minerals is yet to be determined.

Using QEMSCAN to map changes in mineral intensities of the coralline algae skeleton is a new method of examining banding and, due to the high cost of this analysis, and time constraints in this study, only one (long-term) sample was used as a trial in this investigation (S1 Table). The long-term sample used, however, provided highly visual and easily recognisable images of banding that occurs in the coralline skeleton due to slight changes in the mineralogical composition. Skeletal banding in this long-term sample was not obvious when viewed under the more widely used SEM analysis, illustrating the potential application of the QEMSCAN method as another avenue to examine banding in the coralline algae skeleton.

### X-ray Diffraction (XRD) to determine seasonal mineralogy

Seasonal variation in the mineral composition of *P*. *onkodes* from the GBR was observed in our study. The lowest mol% MgCO_3_ was recorded in winter, and the highest in summer at 13.2 and 15.2 mol% MgCO_3_, respectively (Fig 7). This relationship between Mg-calcite composition and seasonal changes in seawater temperature has been reported in many coralline mineralogy studies worldwide (particularly in the higher latitudes), and found to be a useful proxy for determining growth rates and palaeoenvironmental records [17, 19, 20, 27, 62]. However, although seasonal changes in Mg content were observed in the GBR, unlike studies in higher latitudes [17, 20], the range of variation in this study was very low. For example, using an electron microprobe with spot analyses on two rhodolith species, Kamenos *et al*., [17] reported ranges of mol% MgCO_3_ between 12.9–24.6 for *Lithothamion glaciale* and 14.7–23.8 for *Phymatolithon calcareum* in waters off Scotland, and using the same method in the subarctic waters of Newfoundland (Canada), Halfar *et al*., [20] also found large variation in mol% MgCO_3_ ranging from 7.7–18.5 in *L*. *glaciale*. The difference in the seasonal range of variation between the cold and tropical climate studies may be attributed to the different seasonal range of seawater temperature. For example, average seawater temperature varied from 21.8°C– 26.7°C at our study site in the southern GBR [51], while temperate species experienced much larger variability in Scotland (7°C– 16°C) and subarctic Canada (-2°C– 9°C). On the other hand, Moberly [63] compared the Mg content of the encrusting *P*. *onkodes* from the Tuamotu Islands (15.82°S) to *P*. *onkodes* in the warmer waters of Arno Atoll (Marshall Islands, 7.06°S) and found no significant relationship between temperature and Mg content. Moberly [63] pointed out that Mg incorporation was more related to growth rates of *P*. *onkodes* rather than temperature. Our study demonstrated that Mg concentration (and presumably incorporation) was higher in the season of higher average temperature and we established a significant positive relationship between temperature and mol% MgCO_3_ of the epithallus; however high mol% MgCO_3_ did not coincide with the period of maximum vertical growth which occurred in spring [51]. Further experimental studies are needed to identify the drivers governing Mg incorporation and growth of tropical CCA.

The use of different techniques to determine mol% MgCO_3_ may also be a source of variability that could affect comparisons between studies. For example, when comparing measurements from XRD and SEM-EDS analysis Nash *et al*., [48] reported 17.15 mol% MgCO_3_ from the XRD and 15–23 mol% MgCO_3_ from the SEM-EDS. In a separate study Nash *et al*., [8] observed an average composition of 16.78 mol% MgCO_3_ using XRD and a range of 22.60–33.70 mol% MgCO_3_ using ICP-AES (Inductively coupled plasma–atomic emission spectroscopy) (these samples had a varying amount of dolomite and magnesite identified by XRD), highlighting the difficulty in comparing measurements between studies using different methods. Further, Kamenos *et al*., [17] also suggested interspecific variability of Mg incorporation into the calcite skeleton may be a contributing factor to variations in measurements across studies.

## Conclusions

This study has demonstrated that skeletal banding does exist in the tropical encrusting coralline algae species *P*. *onkodes*, and that this banding can be used to provide baseline information into the mineral make-up, growth and reproductive cycles of corallines on the GBR. Seasonal changes in mol % MgCO_3_ were confirmed and appear to follow previously reported variations with the highest mol% MgCO_3_ occurring over summer, and the lowest during winter months. The reproductive cycle was successfully calibrated with the alizarin stain and showed conceptacle banding occurred once a year during the summer period, and that this banding may be a useful proxy for the determination of growth rate of *P*. *onkodes* in other areas of the GBR. Micro-CT scanning provided images of very clear density banding within the coralline algae skeleton that appears to be related to the reproductive cycle. Mineralogical mapping produced images of highly visible banding indicating it also has potential for use in future studies of coralline mineralogy. Applying all these techniques to older *P*. *onkodes* samples may also provide valuable insights into past calcification and growth rates, reproductive patterns, and their variability. For example, since skeletal growth rate and presence of conceptacle bands can be observed uniformly over the past few years, any disruption to these trends can be used to infer environmental and/or climatic variability. This exploratory investigation into the growth, reproductive and mineralogical cycles of a key reef-building species, *P*. *onkodes*, contributes to the small, but growing, body of literature focused on the growth, calcification and life history of coralline algae on the GBR. With the rise of ocean acidification and warming threatening the health of coral reefs, our study is highly pertinent as it will enable the development of metrics that can be used to track the impacts of climate stressors in tropical reefs via the identification of effective, accurate and efficient methods that can be applied to key reef-building organisms.

## Supporting information

S1 Table Crustose coralline algae samples. This Table shows the *Porolithon onkodes* sample used in the analysis of each method.
(PDF)

S1 Data Seasonal mol% Mg of *Porolithon onkodes* using XRD analysis. (XLSX)

S2 Data Seasonal vertical growth of *Porolithon onkodes* measured from alizarin stain. (XLSX)

S3 Data Conceptacle banding measurements using UV images. (XLSX)

S1 Image *Porolithon* onkodes growth banding samples attached to reef. Samples (fragments) of *P*. *onkodes* were set in epoxy rings, and secured to racks on the reef slope at 5–6 m depth in Heron Island reef, Tenements 1. The size of the fragments is 2–3 cm in diameter.
(TIF)

S2 Image Density banding *Porolithon onkodes*. Low resolution X-ray positive image of *P*. *onkodes* sample (PK) indicating density banding is present in the coralline skeleton.
(TIF)

S3 Image Three Micro-CT images (at different density ranges) of the same cross-section showing conceptacles (yellow dots) in the high density bands (light bands) of the *Porolithon onkodes* skeleton. (TIF)
