# Supplementary material for: Presence of skeletal banding in a reef-building tropical crustose coralline alga
Source: PLoS One. 2017 Oct 4;12(10):e0185124. doi: 10.1371/journal.pone.0185124 (PMC5627911; doi:10.1371/journal.pone.0185124)
Supplement: S1 Table — This Table shows the Porolithon onkodes sample used in the analysis of each method. (PDF) [file pone.0185124.s001.pdf]

S2 Table. Crustose coralline algae samples. This Table shows the *Porolithon onkodes* sample used in the analysis of each method.

**Method**	**Purpose**	**Season and sample label**	**Number used**
XRD	Seasonal mol% $MgCO_3$	Spring 2013 – OF, MB, OL, OV, OQ	5 samples per season
		Summer – AR, BR, AV, AQ, AT	
		Autumn – OH, NX, ND, NF, OI	
		Winter – PC, OI, OF, OH, PZ	
X-ray	Density banding	Spring 2013 – OL, OQ, OV	3 samples per season
		Summer – AA, AB, AH	
		Autumn – OK, OL, NV	
		Winter – OL, PL, PK	
Micro-CT	Density banding	Long-term – OE	1 long-term sample
		Spring 2013 – OL	6 seasonal samples
		Summer - AM	
		Autumn – NV	
		Winter – PJ, PK	
		Spring 2014 - LK	
QEMSCAN	Mineralogy banding	Long-term - OE	1 long-term sample
UV Imaging	Conceptacle banding	Long-term OE	1 long-term sample
		Spring 2013 – OL, OQ, OV	3 samples per season

		Summer – AA, AB, AH<br>Autumn – OK, OL, NV<br>Winter – OL, PL, PK	
Alizarin staining	Determine annual and seasonal growth	All samples in study were stained	5 long-term samples<br>20 samples per season
