# Supplementary figures and images for: Presence of skeletal banding in a reef-building tropical crustose coralline alga
Source: PLoS One. 2017 Oct 4;12(10):e0185124. doi: 10.1371/journal.pone.0185124 (PMC5627911; doi:10.1371/journal.pone.0185124)

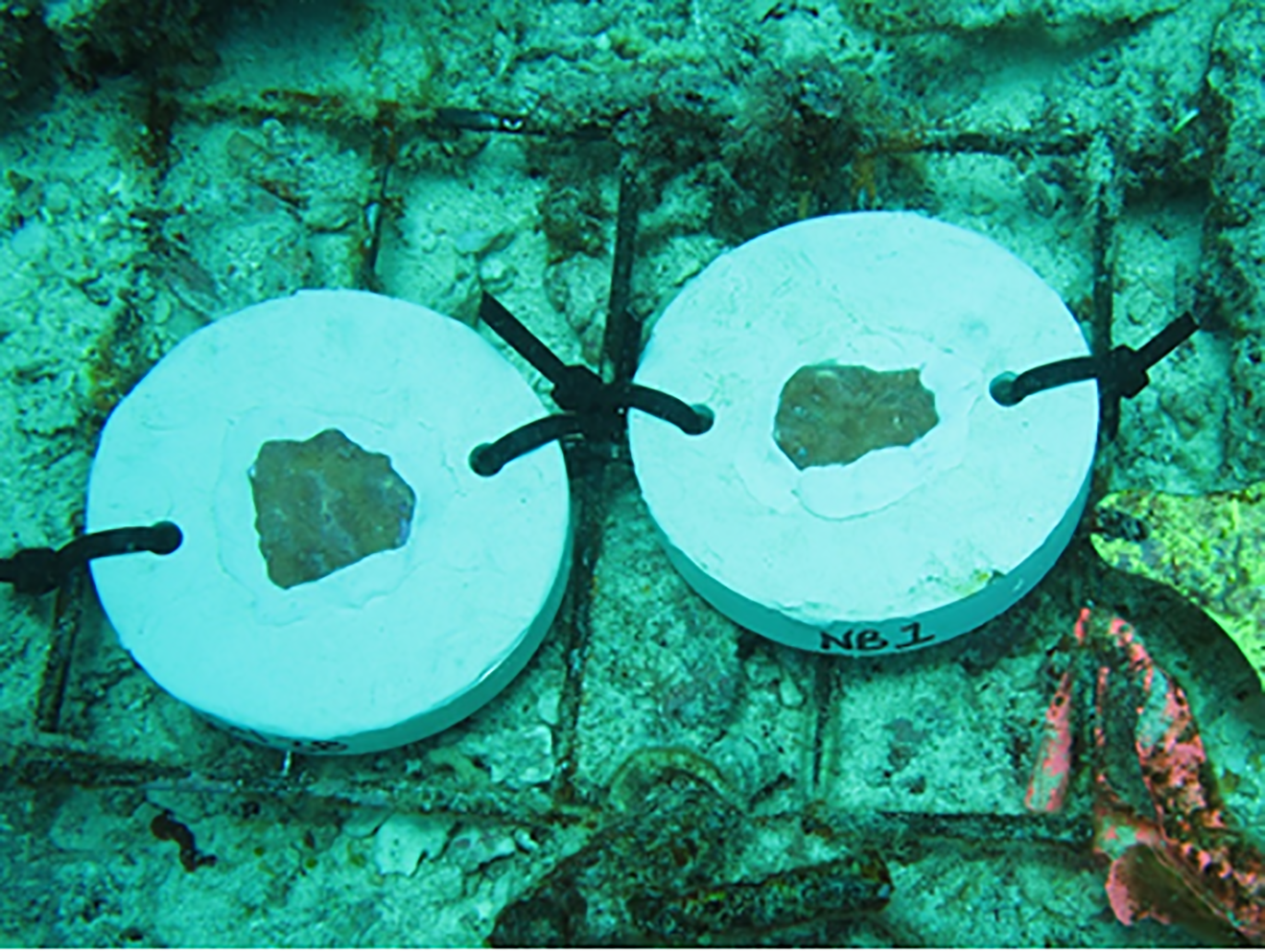

Supplement: S1 Image — Samples (fragments) of P. onkodes were set in epoxy rings, and secured to racks on the reef slope at 5–6 m depth in Heron Island reef, Tenements 1. The size of the fragments is 2–3 cm in diameter. (TIF) [file pone.0185124.s005.tif]

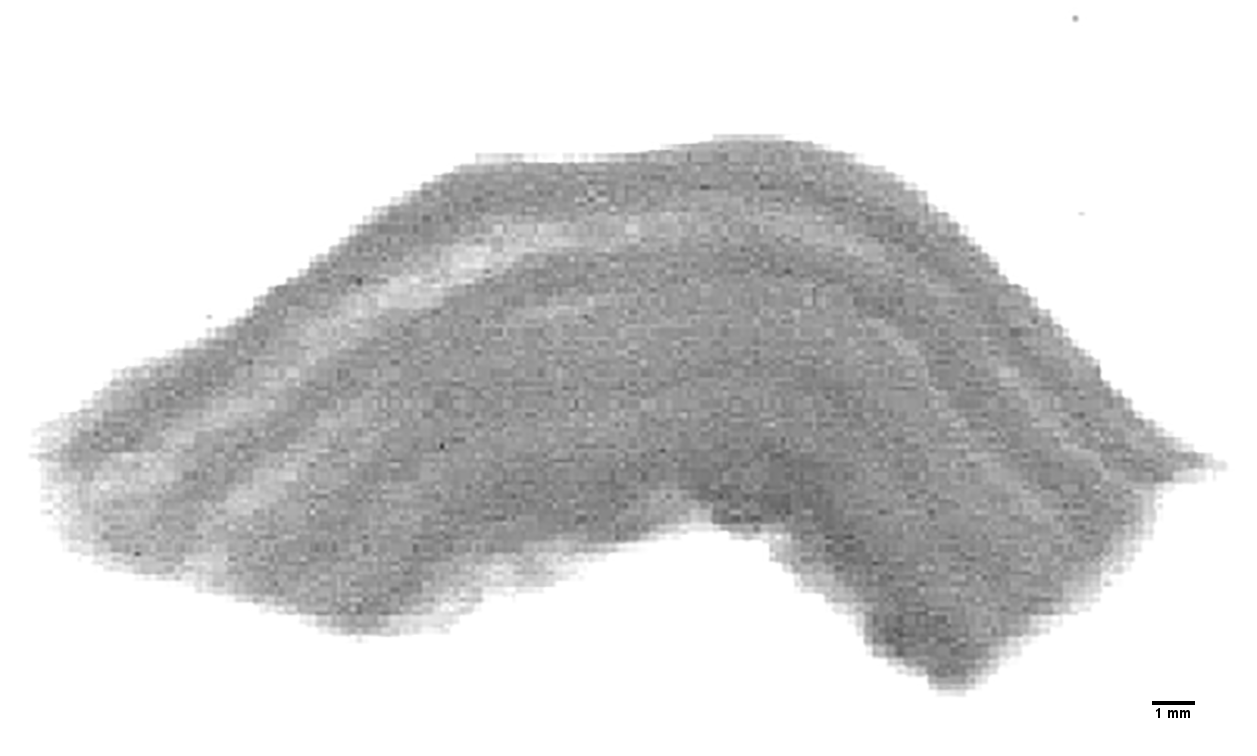

Supplement: S2 Image — Low resolution X-ray positive image of P. onkodes sample (PK) indicating density banding is present in the coralline skeleton. (TIF) [file pone.0185124.s006.tif]

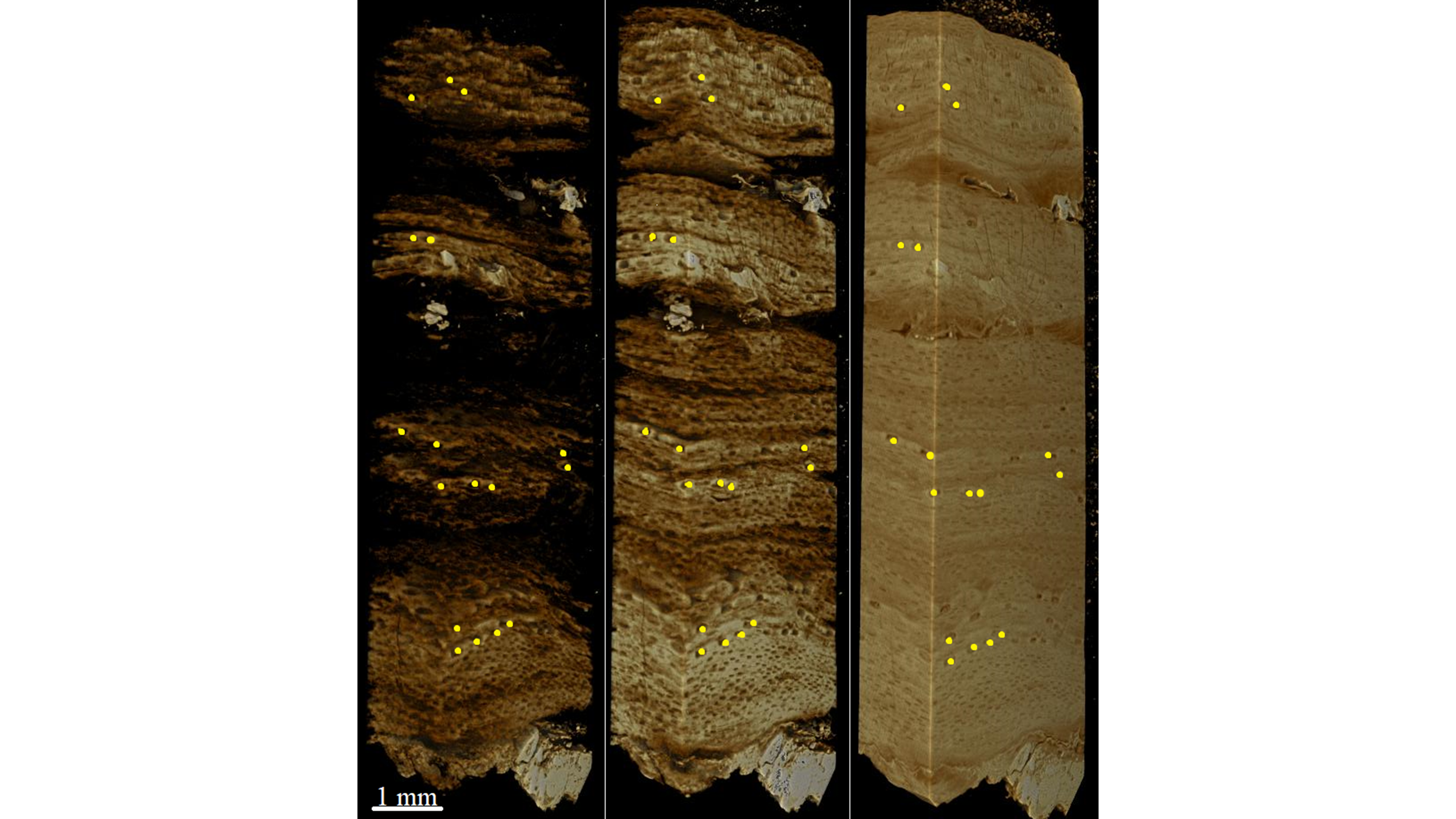

Supplement: S3 Image — (TIF) [file pone.0185124.s007.tif]
